# The Thalamus Regulates Retinoic Acid Signaling and Development of Parvalbumin Interneurons in Postnatal Mouse Prefrontal Cortex

**DOI:** 10.1523/ENEURO.0018-19.2019

**Published:** 2019-03-11

**Authors:** Rachel Larsen, Alatheia Proue, Earl Parker Scott, Matthew Christiansen, Yasushi Nakagawa

**Affiliations:** Department of Neuroscience, University of Minnesota Medical School, Minneapolis, Minnesota 55455

**Keywords:** Cyp26b1, interneurons, parvalbumin, prefrontal cortex, retinoic acid, thalamocortical

## Abstract

GABAergic inhibitory neurons in the prefrontal cortex (PFC) play crucial roles in higher cognitive functions. Despite the link between aberrant development of PFC interneurons and a number of psychiatric disorders, mechanisms underlying the development of these neurons are poorly understood. Here we show that the retinoic acid (RA)-degrading enzyme CYP26B1 (cytochrome P450 family 26, subfamily B, member 1) is transiently expressed in the mouse frontal cortex during postnatal development, and that medial ganglionic eminence (MGE)-derived interneurons, particularly in parvalbumin (PV)-expressing neurons, are the main cell type that has active RA signaling during this period. We found that frontal cortex-specific *Cyp26b1* knock-out mice had an increased density of PV-expressing, but not somatostatin-expressing, interneurons in medial PFC, indicating a novel role of RA signaling in controlling PV neuron development. The initiation of *Cyp26b1* expression in neonatal PFC coincides with the establishment of connections between the thalamus and the PFC. We found that these connections are required for the postnatal expression of *Cyp26b1* in medial PFC. In addition to this region-specific role in postnatal PFC that regulates RA signaling and PV neuron development, the thalamocortical connectivity had an earlier role in controlling radial dispersion of MGE-derived interneurons throughout embryonic neocortex. In summary, our results suggest that the thalamus plays multiple, temporally separate roles in interneuron development in the PFC.

## Visual Abstract

**Figure:**
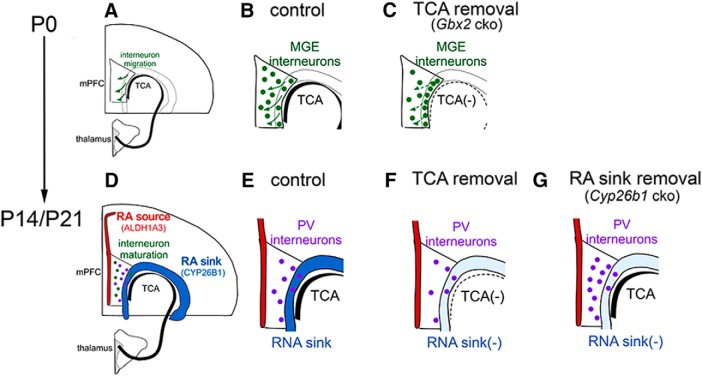

## Significance Statement

Parvalbumin (PV)-expressing inhibitory neurons in the prefrontal cortex (PFC) play a critical role in excitation–inhibition balance and neuronal synchrony during cognitive tasks, and their abnormality is associated with many developmental brain disorders, including schizophrenia and autism. However, molecular and cellular mechanisms for the development of these neurons are not well understood. In this study, we found that retinoic acid (RA) signaling plays an important role in early postnatal development of prefrontal PV neurons, and that the developmentally regulated expression of a key enzyme that restricts RA signaling in the PFC requires the connections between the thalamus and the neocortex. Thus, our results show a novel role of the thalamus in regulating PV neuron development in postnatal PFC.

## Introduction

The prefrontal cortex (PFC) integrates many modalities of information to execute higher functions such as goal-oriented behaviors, social interactions and emotion. Aberrant development of the PFC has been linked to schizophrenia, autism spectrum disorders, attention deficit hyperactivity disorders, depression, and bipolar disorders (Schubert et al., 2015). More specifically, developmental trajectories of GABAergic interneurons in the PFC, particularly those expressing the calcium-binding protein parvalbumin (PV), are impaired in both human patients and animal models of these disorders (Meechan et al., 2012; Nakazawa et al., 2012; Powell et al., 2012; Gonzalez-Burgos et al., 2015; Caballero and Tseng, 2016; Hashemi et al., 2018). Therefore, determining the developmental mechanisms of PFC interneurons is important for understanding the disease pathophysiology.

Development of cortical interneurons is regulated by both intrinsic and extrinsic mechanisms (Bartolini et al., 2013; Chu and Anderson, 2015; Hu et al., 2017; Wamsley and Fishell, 2017). One key extrinsic cue is the input from the thalamus, which regulates the migration and maturation of these neurons (Sugiyama et al., 2008; Marques-Smith et al., 2016; Tuncdemir et al., 2016; Zechel et al., 2016). However, most studies addressing the role of the thalamic input in interneuron development have been performed on primary visual or somatosensory cortex, leaving the mechanisms in PFC understudied. The delayed maturation of PFC interneurons compared with other cortical areas (Gonchar et al., 2007; Nowicka et al., 2009; Ueno et al., 2017) and the distinct set of thalamic nuclei connected with the PFC (Clascá et al., 2012; Nagalski et al., 2016) suggest the presence of unique extrinsic regulatory mechanisms for interneuron development in the PFC.

One candidate molecule that may play a role in postnatal development of the PFC is retinoic acid (RA), a small molecule derived from vitamin A. RA is critical for many important aspects of brain development, ranging from rostrocaudal patterning of the hindbrain and spinal cord to synaptic plasticity (Maden et al., 1996; Dupé and Lumsden, 2001; Molotkova et al., 2007; Chen and Napoli, 2008; Chatzi et al., 2011). The RA-degrading enzyme CYP26B1 (cytochrome P450 family 26, subfamily B, member 1) is crucial in embryonic vertebrate development (Yashiro et al., 2004; Hernandez et al., 2007; Gonzalez-Quevedo et al., 2010). In postnatal mouse neocortex, *Cyp26b1* is expressed in the deep layer of the frontal cortex (Allen Brain Atlas). In addition, *Aldh1a3*, which encodes an RA-synthesizing enzyme, is expressed in the superficial layer of the medial PFC (Wagner et al., 2006). The already established role of RA in embryonic brain development and the unique opposing locations of *Cyp26b1* and *Aldh1a3* expression imply that the balance between the degradation and production of RA might play an unexplored role in postnatal development of medial PFC.

In this study, we demonstrated that in medial PFC, *Cyp26b1* is transiently expressed in layer 6 cells during early maturation of interneurons in the PFC, and that a significant subpopulation of PV interneurons is the main cell type that responds to RA. These results led us to hypothesize that RA signaling regulated by *Cyp26b1* plays a role in controlling the development of PV interneurons in the PFC. To test this, we generated frontal cortex-specific *Cyp26b1* mutant mice and found that these mice had an increased density of PV-expressing neurons in deep layers of medial PFC. We further demonstrated that the postnatal expression of *Cyp26b1* in medial PFC is dependent on the connections between the thalamus and PFC. Thus, the thalamus has a postnatal role in regulating the development of PV neurons via inducing the expression of the RA-degrading enzyme CYP26B1 in frontal cortex. Additionally, we found that the thalamus is also required for the radial allocation of PFC interneurons during embryogenesis. We therefore propose that the thalamus plays multiple crucial roles in interneuron development in the PFC first by controlling their laminar positioning during embryonic stages, and thereafter by restricting the maturation of PV neurons through the induction of a retinoic acid-degrading enzyme.

## Materials and Methods

### Mice

*RARE-LacZ* transgenic mice (Rossant et al., 1991) were obtained from The Jackson Laboratory (stock #008477) and were kept in the CD1 background. Frontal cortex-specific *Cyp26b1* mutant mice were generated using BAC (bacterial artificial chromosome) *Syt6-Cre* mice (GENSAT; Gong et al., 2003; Hsu et al., 2014). Although the endogenous *Syt6* (*Synaptotagmin 6*) gene is expressed ubiquitously in layer 6 of the neocortex, the BAC Cre line caused recombination specifically in the frontal cortex. We crossed *Syt6^Cre/+^; Cyp26b1^flox/+^* mice and *Cyp26b1^flox/flox^* mice to generate the conditional mutants. *Cyp26b1^flox/flox^* mice (Okano et al., 2012) were developed by Dr. Hiroshi Hamada’s laboratory (RIKEN Center for Developmental Biology, Kobe, Japan) and obtained from Dr. Maria Morasso (National Institute of Arthritis and Musculoskeletal and Skin Diseases, Bethesda, MD). *Rosa26^stop-ZSGreen/+^* (*Ai6*) mice (Madisen et al., 2010) were obtained from The Jackson Laboratory (stock #007906). Thalamus-specific *Gbx2* mutant mice were generated by crossing *Olig3^Cre/+^; Gbx2^null/+^* mice and *Gbx2^flox/flox^* mice as described previously (Vue et al., 2013). *Gbx2^flox/flox^* mice were obtained from The Jackson Laboratory (Li et al., 2002). *Olig3^Cre/+^*mice were described previously (Vue et al., 2009; Bluske et al., 2012). The *Gbx2^null^* allele was generated by crossing *Gbx2^flox/flox^* mice with the *CMV-Cre* germline deleter mice (stock #003465, The Jackson Laboratory). Mice that express tetanus toxin light chain (TeNT) in thalamic neurons were generated by crossing *Olig3^Cre/+^* mice and *Rosa26^stop-TeNT/stop-TeNT^* mice (Zhang et al., 2008).

### *In situ* hybridization

cDNAs for the following genes were used: *Cyp26b1*, *Aldh1a3*, *Syt6*, and *Lmo4* (Open Biosystems); *Pvalb*, *Sst*, and *Vip* (obtained from Dr. Rob Machold, New York University); *Lhx6* (obtained from Dr. John Rubenstein, University of California San Francisco); and *RORβ* (obtained from Dr. Michael Becker-Andre, Ludwig Maximilians University of Munich). Postnatal pups were perfused with 4% paraformaldehyde (PFA)/0.1 m phosphate buffer, and the heads were postfixed until needed. Brains were then taken out of the skull, washed in 0.1 m phosphate buffer for 20 min and were sunk in 30% sucrose/0.1 m phosphate buffer. Coronal sections were cut with a sliding microtome at 50 µm thickness (Leica) or with a cryostat at 20 µm [postnatal day 2 (P2) or younger] or 40 µm (P4 or older) thickness and were mounted on glass slides (Super Frost Plus, Thermo Fisher Scientific). *In situ* hybridization was conducted as described previously (Vue et al., 2007).

### Immunohistochemistry

Brains were taken out immediately after perfusion and were postfixed for 1 h (P0), 1–2 h (P4–P14) or 1–4 h (P21). After the postfixation, the brains were washed in 0.1 m phosphate buffer for 20 min and were sunk in 30% sucrose/0.1 m phosphate buffer. Sections were cut as described above for *in situ* hybridization. The following primary antibodies were used: β-galactosidase (β-gal; 1:100, goat, catalog #55976, Cappel; 1:500, chicken, catalog #ab9361, Abcam); SOX6 (1:100, rabbit, catalog #ab30455, Abcam); SP8 (1:100, goat, catalog #sc-104661, Santa Cruz Biotechnology); CTIP2 (1:200, rat, catalog #ab18465, Abcam); TBR1 (1:200, rabbit, catalog #ab31940, Abcam; 1:200, chicken, catalog #AB2261, Millipore); PV (1:500, rabbit, catalog #PV27, SWANT); somatostatin (SST; 1:100, rat, catalog #MAB354, Millipore); LIM Homeobox 6 (LHX6; 1:50, mouse, catalog #sc-271433, Santa Cruz Biotechnology); vesicle-associated membrane protein 2 (VAMP2; 1:200, rabbit, catalog #104 202, Synaptic Systems); NetrinG1 (1:100, goat, catalog #AF1166, R&D Systems); and cleaved caspase 3 (1:100, rabbit, catalog #D175, Cell Signaling Technology). Secondary antibodies conjugated with Cy2, Cy3, or Cy5 were obtained from Jackson ImmunoResearch.

### Combined *in situ* hybridization (*Sst*) and immunohistochemistry (SOX6)

*In situ* hybridization was conducted as described above, except that before proteinase K treatment, sections were incubated with 0.3% hydrogen peroxide/PBS for 10 min to block endogenous peroxidase activity. After the hybridization, slides were washed as described above, and were incubated for 30 min with anti-digoxigenin antibody conjugated with peroxidase, followed by two washes in PBS and one wash with TNT solution (TNT: 0.1 m Tris, pH 7.5, 0.15 m NaCl, 0.05% Tween 20). Sections were then incubated for ∼30 min in Tyramide Plus Fluorescein (Thermo Fisher Scientific) diluted at 1:250, washed twice in TNT solution, and postfixed. Thereafter, immunostaining with anti-SOX6 antibody was conducted as described above.

### Imaging and binning

**Figure 1.**
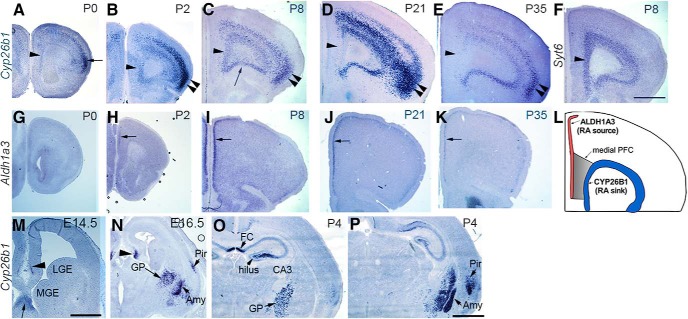
Spatiotemporally regulated expression of *Cyp26b1* and *Aldh1a3* in the PFC and other forebrain regions. *In situ* hybridization of frontal sections through PFC at various stages is shown. ***A–E***, *Cyp26b1* expression in frontal cortex. At P0, only a weak expression is seen in medial PFC (***A***, arrowhead). ***B***, At P2, *Cyp26b1* starts to be detected clearly in medial PFC (***B***, arrowhead); expression in lateral cortex, especially agranular insula in more superficial layer (***A***, arrow) is strong, which continues into later stages (***B–E***, double arrowheads). At P8, expression in medial (***C***, arrowhead) and ventral (***C***, arrow) PFC is strong. At P21, the expression of *Cyp26b1* is reduced in medial PFC (***D***, arrowhead) and is almost undetectable by P35 (***E***, arrowhead). ***F***, *Syt6*, a layer 6 marker, is expressed in the same layer as *Cyp26b1* in medial PFC at P8 (arrowhead). ***G–K***, *Aldh1a3* expression in frontal cortex. At P0, expression is not detected in PFC (***G***). At P2, clear expression is detected in the superficial layer of medial PFC (***H***, arrow). The expression continues into P8, P21, and P35 (***I–K***, arrow). ***L***, Schematic summary of the spatial expression patterns of *Cyp26b1* and *Aldh1a3* in medial PFC of early postnatal mouse brains. ***M***, Expression of *Cyp26b1* is not detected in LGE or MGE at E14.5, but is already found in the hippocampus (arrowhead), septum (arrow), globus pallidus (data not shown), and amygdala (data not shown). ***N***, At E16.5, *Cyp26b1* is detected in hippocampus (arrowhead), piriform cortex (Pir), globus pallidus (GP), and amygdala (Amy). ***O***, ***P***, This pattern continues into P4 and adulthood (data not shown). ***P*** is at a more caudal level than ***O***. Expression in the hippocampus is strongest in CA3 and hilus, whereas multiple nuclei in amygdala show strong expression of *Cyp26b1* (***P***). Scale bars: ***A–K***, ***N–P***, 1 mm; ***M***, 500 μm.

**Figure 2.**
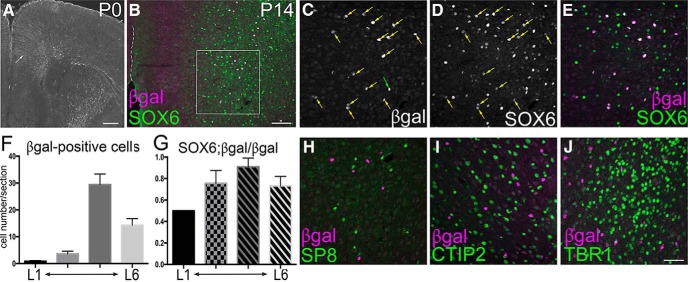
RA signaling in early postnatal PFC. In all sections, the right hemisphere is shown, and the white dashed line marks the medial surface of the frontal cortex. ***A–E***, ***H–J***, Immunostaining for β-gal on frontal sections of P0 (***A***) and P14 (***B–E***, ***H–J***) brains of *RARE-LacZ* transgenic mice. ***A***, At P0, β-gal expression is found only in the radial glial fibers (arrow). ***B–J***, At P14, β-gal-positive cells are abundant in medial PFC and they are SOX6 positive. ***B*** is a 10× image including the medial surface of the brain, and ***C–E*** are 40× confocal images of the same region outlined in the square in ***B***. ***E*** is the merged image of ***C*** and ***D***. Yellow arrows in ***C*** and ***D*** show SOX6/β-gal-double-positive cells, and green arrow in ***C*** shows a rare, β-gal-positive, SOX6-negative cell. ***F***, Average number of β-gal-positive cells per section by layers (mean ± SEM). L1, Layer 1 as marked by sparse labeling in DAPI staining; L6, layer 6 as marked by TBR1 staining. The two middle columns represent equal-width bins between layer 1 and layer 6, and approximately corresponds to layers 2/3 and layer 4/5, respectively. Because most β-gal-positive cells are immediately above layer 6, and layer 4 is thin in medial PFC (Fig. 6*G*), the highest peak in the third column likely represents layer 5. ***G***, The ratios of SOX6; β-gal-double-positive cells among β-gal-positive cells are shown by layers (mean ± SEM). ***H–J***, β-gal does not overlap with SP8, CTIP2, or TBR1. Scale bars: ***A***, 200 μm; ***B***, 100 μm; ***C–E***, ***H–J***, 50 μm.

**Figure 3.**
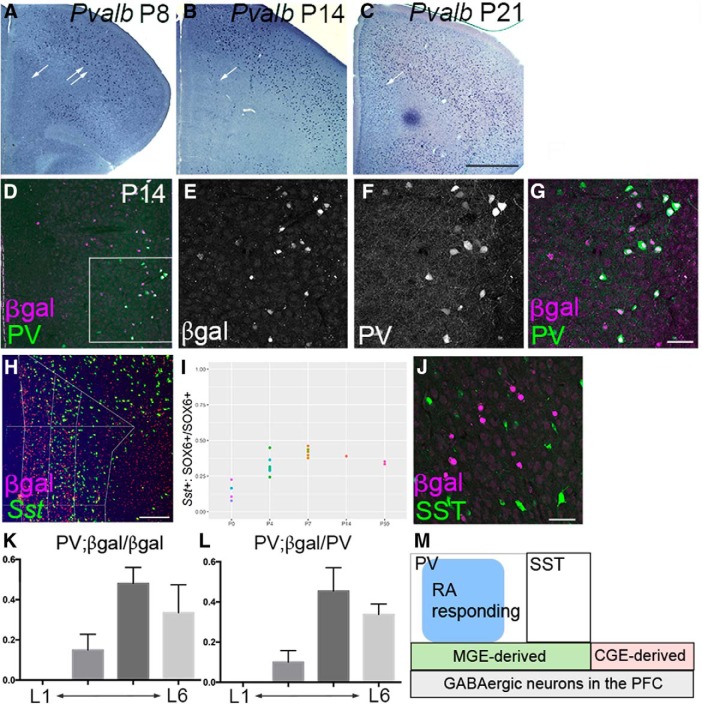
Overlapping expression of *RARE-LacZ* transgene and PV in early postnatal PFC. ***A–C***, *In situ* hybridization for *Pvalb* mRNA on frontal sections of control mice at P8 (***A***), P14 (***B***), and P21 (***C***). In medial PFC, *Pvalb* is undetectable at P8, but is robustly expressed at P14, which further increases by P21 (***A–C***, single arrow). *Pvalb* mRNA is already expressed in many cells in dorsolateral frontal cortex (***A***, double arrow). ***D–G***, Double immunostaining for β-gal and PV on frontal sections of P14 brains of *RARE-LacZ* transgenic mice. ***D*** is a 10× image including the medial surface of the brain, and ***E–G*** are 40× confocal images of the same region outlined in the square in ***D***. ***G*** is a merged image of ***E*** and ***F***. Note the heavy overlap between β-gal and PV. ***H***, ***I***, Timecourse of *Sst* expression in medial PFC of postnatal mice. In ***H***, *Sst* mRNA was detected by *in situ* hybridization using a Tyramide Signal Amplification system, followed by immunostaining with anti-SOX6 antibody. The section is from a control P14 PFC and left is to the medial surface. In ***I***, the ratio of *Sst*-positive, SOX6-positive cells to SOX6-positive cells in medial PFC is shown for P0, P4, P7, P14, and P59. A plateau value of ∼0.4 is reached by P7. At P0, a much smaller portion of SOX6-positive cells expressed *Sst* mRNA. Each dot indicates an average number of cells in medial PFC of at least three sections of a wild-type brain. ***J***, Double immunostaining for β-gal and SST on frontal sections of P14 brains of *RARE-LacZ* transgenic mice. Note the little or no overlap between β-gal and SST. ***K***, The ratios of PV; β-gal-double-positive cells among β-gal-positive cells in P14 medial PFC are shown by layers (mean ± SEM). ***L***, The ratios of PV/β-gal-double-positive cells among PV-positive cells in P14 medial PFC are shown by layers (mean ± SEM). ***M***, A schematic summary of the interneuron populations in medial PFC. Based on the results of this study, a subpopulation of PV interneurons responds to RA via RAR/RXR receptor complex. Scale bars: ***A–C***, 1 mm; ***D***, 100 μm; ***E–G***, ***J***, 50 μm; ***H***, 200 μm.

For cell counting, images of sections that underwent *in situ* hybridization or immunostaining were taken using an upright microscope (model E800, Nikon) with a 2× (*in situ* hybridization) or a 4× (immunostaining) objective using a digital CCD camera (Retiga EXi, QImaging) and the OpenLab software. Coronal sections of the rostral cortex that contain the forceps minor (anterior forceps) of the corpus callosum in the center (Fig. 1*A–F*) were used for analysis. Sections in which the forceps minor is extending toward the medial surface as well as the sections that include the corpus callosum itself were excluded as being too caudal. Once the sections were selected for each brain, putative prelimbic and infralimbic areas of the medial PFC (Allen Brain Atlas; Paxinos et al., 2006; Van De Werd et al., 2010) were binned for cell counting. As seen in Figure 5, *A* and *B*, three dorsal–ventral bins were drawn using Photoshop CS5. Each bin is 500 µm high at the medial surface. Each of these three bins was further subdivided into smaller bins by their laminar locations; for sections with *in situ* hybridization, the most superficial layer (layer 1) was defined as the cell–sparse layer on DAPI staining. The remaining cortical wall was divided into three bins with equal widths, resulting in the total of four laminar bins. Layer 1 and the layer underneath it were grouped together and were named superficial layers, and the remaining two layers were named the deep layers. For immunostaining, we used anti-TBR1 (T-Box, Brain 1) antibody for all slides in Cy5 channel and used it as a reference marker of layer 6. Then, the areas excluding layers 1 and 6 were equally divided into three parts, resulting in five laminar bins (see Fig. 7). For Figures 2, *F* and *G*, and 3, *K* and *L*, the portion between layer 1 and layer 6 was divided into two equal parts, resulting in four laminar bins. For high-magnification images shown in Figures 2 and 3, an Olympus FluoView 1000 confocal microscope was used (40× oil; numerical aperture, 1.25).

### Cell counting

Using the ImageJ or Fiji program, the original color images were converted to inverted black and white images, and dark spots were automatically counted by the ITCN (Image-based Tool for Counting Nuclei) plugin. Cell density was calculated by measuring the area of each bin and dividing the cell number by the area of each bin. At least two sections per brain were used for cell counting, and the numbers were averaged to represent the brain. Two brains (mutant and wild type) from the same litter that were processed and analyzed in the same experiment were compared as a pair.

### Experimental design and statistical analysis

Both males and females were used in this study. A paired ratio *t* test was used for comparing cell counts between *Cyp26b1* CKO (conditional knock-out) mice and wild-type littermates as well as *Gbx2* CKO and wild-type littermates. For data seen in Figures 5 and 7, we also performed repeated-measures two-way ANOVA using Prism (versions 6 and 7, GraphPad Software). Graphs were generated using Prism.

### Axon tracing

Small crystals of 1,1´-dioctadecyl-3,3,3´3´-tetramethylindocarbocyanine perchlorate (DiI) were placed on the medial surface of frontal cortex of PFA-fixed P14 *Gbx2* conditional mutant brains and their control littermates. After incubation of the brains in PFA at 37°C for 2 weeks, we cut sections at 150 µm with a vibrating microtome (Oscillating Tissue Slicer, Electron Microscopy Sciences), counterstained the sections, and mounted them on glass slides for imaging.

## Results

### *Cyp26b1,* a gene encoding a retinoic acid-degrading enzyme, is expressed in a spatially and temporally dynamic pattern in postnatal mouse PFC

To identify genes that are enriched in developing PFC, we screened the Anatomic Gene Expression Atlas (AGEA; Ng et al., 2009). The database showed that *Cyp26b1*, a gene that encodes a retinoic acid (RA)-degrading enzyme that belonged to the cytochrome P450 family 26 (CYP26) proteins, is strongly expressed in the frontal cortex at P14, including the deep layer of medial and ventral PFC as well as the middle layer of lateral cortex extending into the agranular insula. We therefore examined the developmental expression patterns of *Cyp26b1* in more detail by *in situ* hybridization. Prenatal cortex did not show detectable *Cyp26b1* expression (data not shown). At P0, lateral frontal cortex, but not medial PFC, started to show a robust signal (Fig. 1*A*). At P2, strong expression of *Cyp26b1* was detected in medial PFC, as well as in the dorsolateral frontal cortex including the motor area (Fig. 1*B*). Comparison with the established marker of layer 6 neurons, *Syt6*, showed that *Cyp26b1* is expressed in layer 6 of medial and ventral PFC (Fig. 1, compare *C* and *F*). Not only was the expression of *Cyp26b1* spatially restricted, it was also temporally dynamic; in medial PFC, *Cyp26b1* was strong at P8 (Fig. 1*C*) and P14 (see Fig. 6*M*). However, by P21, *Cyp26b1* expression in medial PFC was much weaker compared with ventral and lateral regions (Fig. 1*D*). By P35, there was no detectable expression of *Cyp26b1* in medial PFC (Fig. 1*E*).

**Figure 4.**
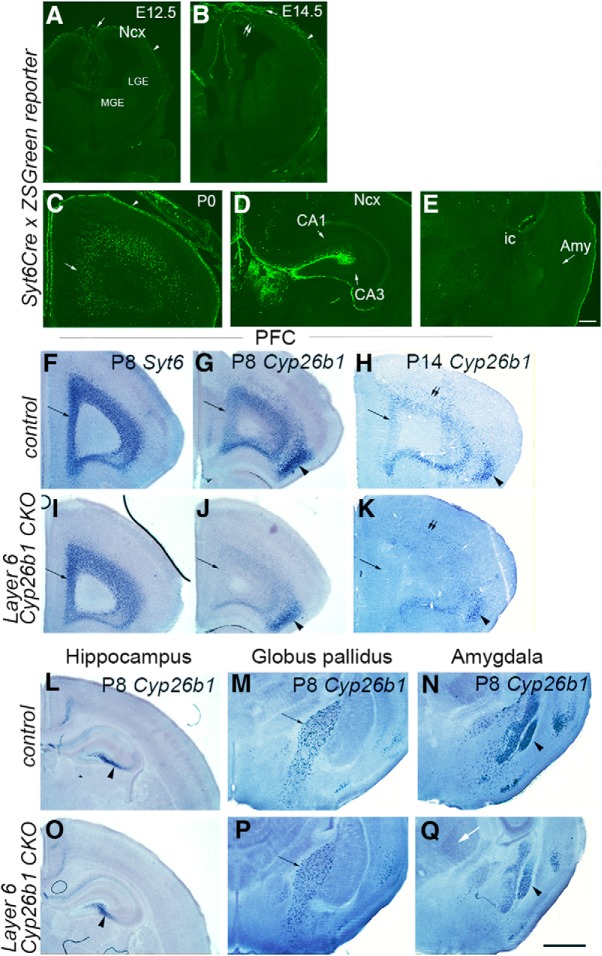
Conditional deletion of *Cyp26b1* using *Synaptotagmin6-Cre* (*Syt6-Cre*). ***A–E***, Recombination in *Syt6-Cre* transgene mice. ***A–E***, Expression of ZSGreen in *Syt6-Cre/+*; *Ai6* (ZSGreen Cre reporter) mice at E12.5 (***A***), E14.5 (***B***), and P0 (***C–E***) are shown. All sections are coronal, and the midline is to the left. At E12.5, the expression of ZSGreen reporter is found in meninges (***A***, arrow) and preplate (***A***, arrowhead), but not in the rest of the cortex or MGE and LGE. At E14.5, a small number of cortical cells (***B***, double arrows) below the marginal zone (***B***, arrowhead) start to express ZSGreen. ***C–E***, At P0, many layer 6 cells of frontal cortex express ZSGreen (***C***, arrow), but not in more caudal neocortex (***D***, Ncx), CA1, CA3, and hilus regions of the hippocampus (***E***, note that strong signal is found in the meninges of the hippocampus) or the amygdala (***F***, Amy). ic, Internal capsule. Scale bar, 200 μm. ***F–Q***, Generation of conditional *Cyp26b1* knock-out mice. ***F–Q***, *In situ* hybridization of frontal sections of P8 (***F***, ***G***, ***I***, ***J***, ***L–Q***) or P14 (***H***, ***K***) *Cyp26b1* conditional knock-out (***I–K***, ***O–Q***) and control littermate (***F–H***, ***L–N***) brains. *Cyp26b1* was conditionally knocked out using the *Syt6-Cre* transgene. *Syt6* is expressed in layer 6 of both control (***F***) and *Cyp26b1* knock-out (***I***) brains (arrow). The expression of *Cyp26b1* in layer 6 of frontal cortex (arrow) is detected in control brains, but not in *Cyp26b1* knock-out brains at P8 (***G***, ***J***) and P14 (***H***, ***K***). The expression of *Cyp26b1* in agranular insula is unchanged in *Cyp26b1* knockouts (***G***, ***H***, ***J***, ***K***, arrowhead). The expression of *Cyp26b1* in CA3 and hilus region of the hippocampus (***L***, ***O***, arrowhead), globus pallidus (***M***, ***P***, arrow), and amygdala (***N***, ***Q***, arrowhead) is unchanged in *Cyp26b1* knockouts. Scale bar, 1 mm.

The tissue RA level is controlled both by its synthesis from vitamin A and by its degradation by CYP26 enzymes. Members of the aldehyde dehydrogenase 1 (ALDH1) family are crucial for synthesizing RA, and two members of this family (ALDH1A2 and ALDH1A3) are expressed in early postnatal cortex; *Aldh1a2* is broadly expressed in the meninges (Wagner et al., 2002), whereas *Aldh1a3* is specifically expressed in the superficial layer of postnatal medial PFC as well as higher-order visual areas (Wagner et al., 2002, 2006). We found that the expression of *Aldh1a3* becomes detectable in medial PFC at P2 (Fig. 1*H*). By P8, the expression was robust in layer 2 and extended more laterally (Fig. 1*I*). A similar pattern of expression was found at P21 (Fig. 1*J*) and P35 (Fig. 1*K*). There also appears to be *Aldh1a3* expression in medial cortex in adult mice (Allen Brain Atlas). Outside of the frontal cortex, we did not detect *Cyp26b1* expression in more caudal parts of the neocortex including sensory areas at any developmental stage examined (Figs. 1*N*,*O*, 4*L*). *Cyp26b1* was also expressed in the piriform cortex, the amygdala, CA3 and the hilus regions of the hippocampus, and the globus pallidus (Fig. 1*N–P*). Expression in these regions started during embryogenesis (Fig. 1*N*) and continued into adulthood (Allen Brain Atlas). *Cyp26b1* was not detected in either medial ganglionic eminence (MGE) or lateral ganglionic eminence (LGE; Fig. 1*M*).

In summary, in the medial part of early postnatal PFC, RA is produced by cells in the superficial layer, whereas CYP26B1, an RA-degrading enzyme or “RA sink,” is located in layer 6 (Fig. 1*L*). These results suggest that RA signaling is spatially and temporally controlled, and that this regulation might play a role in the development of medial PFC.

### Parvalbumin-expressing interneurons in medial PFC respond to retinoic acid during early postnatal development

The spatiotemporal expression pattern of *Cyp26b1* prompted us to explore the cellular targets of RA signaling during postnatal PFC development. To determine the populations of cells that respond to RA, we analyzed the expression of the *RARE-LacZ* transgene, an indicator of the transcriptional activity of the RA receptor RAR (retinoic acid receptor)/RXR (retinoid X receptor) heterodimers (Rossant et al., 1991). At P0, the expression of β-gal was barely detectable in medial PFC except in radial glial fibers (Fig. 2*A*). At P14, a robust population of β-gal-positive cells was detected in medial PFC, mostly in layer 5. These cells were also positive for SOX6, a marker for GABAergic interneurons derived from MGE (Batista-Brito et al., 2009; Fig. 2*B–F*). Analysis of three transgenic brains revealed that 91% of β-gal-positive cells in layer 5 were also SOX6 positive (Fig. 2*G*). Markers of other neuronal types, including SP8 [Fig. 2*H*; caudal ganglionic eminence (CGE)-derived cortical interneurons; Ma et al., 2012], CTIP2 (Fig. 2*I*; layer 5 subcerebral projection neurons as well as some interneurons; Arlotta et al., 2005; Chen et al., 2005; Nikouei et al., 2016), and TBR1 (Fig. 2*J*; layer 6 corticothalamic projection neurons; Hevner et al., 2001) did not overlap with β-gal, indicating that these types of neurons do not express the molecular machinery for responding to RA via RAR/RXR heterodimers. In summary, MGE-derived interneurons in layer 5 are the main responders to RA within early postnatal medial PFC.

Most MGE-derived interneurons in the adult neocortex express either SST or PV (Fogarty et al., 2007; Miyoshi et al., 2007). A majority of these interneurons complete their tangential migration into the neocortex by birth (Miyoshi and Fishell, 2011; Inamura et al., 2012). However, PV protein or *Pvalb* mRNA is not expressed until much later, suggesting that the expression of PV or *Pvalb* is a useful marker of maturation for this lineage of cells. At P8, *Pvalb* mRNA was already abundant in the lateral portion of the neocortex including motor area (Fig. 3*A*, double arrows), but not in medial PFC (Fig. 3*A*, arrow). Thus, maturation of PV neurons is delayed in medial PFC. By P14, cells expressing *Pvalb* mRNA or PV protein were clearly detectable in medial PFC, mainly in layer 5 (Fig. 3*B*, arrow), which further increased by P21 (Fig. 3*C*, arrow).

To determine whether RA-responding cells are restricted to either PV or SST neurons, we next analyzed *RARE-LacZ* transgenic mice to determine the colocalization of β-gal and PV (Fig. 3*D–G*) as well as β-gal and SST (Fig. 3*J*) at P14. We found a heavy overlap between PV and β-gal; in three P14 brains (Fig. 3*D–G*), 48% percentage of β-gal-positive cells in layer 5 were also PV positive (Fig. 3*K*). In turn, 45% of PV-positive cells in layer 5 were also β-gal-positive (Fig. 3*L*). Similar patterns were found at P21 (data not shown). In a sharp contrast, SST showed very little overlap with β-gal from P8 through P67 (Fig. 3*J*; and data not shown); the ratio of SST/β-gal double-positive cells to SST-positive cells was 2.9% (3 of 103) at P14 and 5.0% (6 of 121) at P21. In medial PFC, cells expressing SST protein or *Sst* mRNA appeared between P0 and P4; by P7, ∼40% of MGE-derived interneurons that express SOX6 also expressed *Sst* mRNA, and this ratio stayed constant until P59 (Fig. 3*H*,*I*). Thus, by P7, most SST neurons in medial PFC already express *Sst* mRNA, and MGE-derived interneurons that are β-gal-positive and SST-negative are most likely to be PV neurons (Fig. 4*M*).

### *Cyp26b1* is required for normal development of parvalbumin-expressing interneurons in medial PFC

Because of the strong correlation between the expression of PV and the responsiveness of the cell to RA, we hypothesized that the development of PV interneurons in medial PFC is regulated by RA signaling and that this regulation depends on CYP26B1. To test this, we generated conditional *Cyp26b1* mutant mice in which *Cyp26b1* is deleted in the PFC. Because the expression of *Cyp26b1* is highly specific to layer 6 in the frontal cortex, we used *Syt6-Cre* driver mice (Gong et al., 2003; Hsu et al., 2014). Expression *Syt6* is specific to layer 6 in the neocortex (Fig. 1*F*), and *Syt6-Cre* mice allow recombination in layer 6 corticothalamic projection neurons in the frontal cortex including the medial PFC (Allen Brain Atlas; http://connectivity.brain-map.org/). To validate the usefulness of *Syt6-Cre* in knocking out *Cyp26b1* in layer 6 of the postnatal frontal cortex but not in other, potentially relevant *Cyp26b1*-expressing cell populations, we first bred the Cre mice with ZSGreen Cre reporter mice (Ai6; Madisen et al., 2010). At embryonic day 12.5 (E12.5), *Syt6-Cre*; *Ai6* brains showed ZSGreen expression in the preplate and the meninges but not in other parts of the cortex or in ganglionic eminences (Fig. 4*A*). Cortical expression of ZSGreen started to be detected at E14.5 (Fig. 4*B*). At P0, robust signs of recombination were seen in layer 6 of the frontal cortex but not in more caudal cortex (Fig. 4*C*,*D*), amygdala (Fig. 4*E*), or CA3 and hilus regions of the hippocampus (Fig. 4*D*). Thus, we predicted that the *Syt6-Cre* mice would cause specific deletion of *Cyp26b1* in layer 6 cells of the frontal cortex including medial PFC. In *Syt6-Cre/+*; *Cyp26b1^flox/flox^* (*Cyp26b1 CKO*) mice, *Cyp26b1* mRNA was not detected in medial PFC (Fig. 4, compare *G*, *J* and *H*, *K*), whereas expression in other brain regions including the hippocampus (Fig. 4*L*,*O*), agranular insula (Fig. 4*G*,*J*), globus pallidus (Fig. 4*M*,*P*), or amygdala (Fig. 4*N*,*Q*) was not affected, confirming an efficient and specific deletion of *Cyp26b1*.

**Figure 5.**
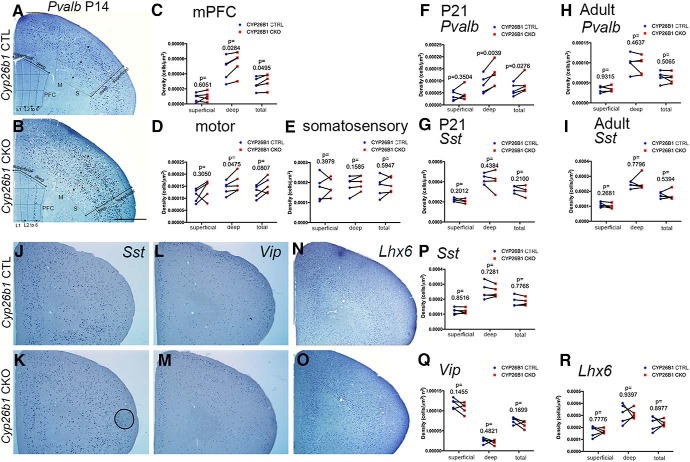
Increased *Pvalb*-expressing interneurons in medial PFC of *Cyp26b1* knock-out mice. ***A***, ***B***, *In situ* hybridization of frontal sections of P14 *Cyp26b1* conditional knock-out mice (***B***) and littermate controls (***A***). Expression of *Pvalb* mRNA is shown. See Materials and Methods on the binning of the medial PFC. Numbers of *Pvalb*-positive cells in the two superficial bins and two deep bins were added together and compared separately between *Cyp26b1* mutants and littermate controls. ***C–E***, Result of paired ratio *t* tests for cell counts in the medial PFC (***C***), motor cortex (***D***), and somatosensory cortex (***E***), all on the same frontal sections. Each line connecting red and blue dots represents a pair of brains analyzed in the same experiment (*n* = 5). The *p* values of the ratio of paired *t* tests for each layer (superficial, deep, total) are shown. In repeated-measures two-way ANOVA, the *p* values for layer (superficial versus deep), pair (between control and knockout), and interactions (between layer and pair) are 0.0017, 0.0325, and 0.1416 (P14 in PFC); 0.0441, 0.0961 and 0.7807 (P14 in motor cortex); 0.1771, 0.4751, and 0.5496 (P14 in somatosensory cortex), respectively. Scale bar, 1 mm. L1, layer 1. ***F***, ***G***, At P21, the density of *Pvalb*-expressing cells (***F***), but not *Sst*-expressing cells (***G***), is increased in *Cyp26b1* conditional knock-out mice. In repeated-measures two-way ANOVA, the *p* values for layer (superficial versus deep), pair (between control and knockout), and interactions (between layer and pair) are 0.0047, 0.0287, and 0.0637 (*Pvalb*); 0.0065, 0.3621, and 0.7609 (*Sst*). ***H***, ***I***, No significant changes in the density of *Pvalb*- and *Sst-*expressing interneurons in medial PFC of adult (P56–P67) *Cyp26b1* knock-out mice. Each line connecting red and blue dots represents a pair of brains analyzed in the same experiment (*n* = 4). The *p* values of paired *t* tests for individual layers are shown. ***J–R***, No significant changes in the number of *Sst-*, *Vip-*, and *Lhx6-*expressing interneurons in medial PFC of *Cyp26b1* knock-out mice at P14. ***J–O***, *In situ* hybridization of frontal sections of P14 *Cyp26b1* conditional knock-out mice (***J***, ***L***, ***N***) and littermate controls (***K***, ***M***, ***O***). Expression of *Sst* (***J***, ***K***), *Vip* (***L***, ***M***), and *Lhx6* (***N***, ***O***) is shown. Binning and cell counts were performed as shown in ***A*** and ***B***. Scale bar, 1 mm. ***P–R***, Result of statistical analysis. Each line connecting red and blue dots represents a pair of brains analyzed in the same experiment (*n* = 5). The *p* values of paired ratio *t* test for individual layer are shown.

We then counted *Pvalb* mRNA-expressing neurons and compared the numbers between *Cyp26b1* CKO mice and their littermate wild-type controls at P14 and P21 (Fig. 5). At both stages, the density of *Pvalb*-positive cells was significantly increased in medial PFC of *Cyp26b1* mutant mice (Fig. 5*C*,*F*, “total”). When the PFC was divided into superficial and the deep layers, the difference was seen only in the deeper half of the medial PFC, consistent with the distribution of β-gal-expressing cells. With repeated-measures two-way ANOVA, we also detected significant differences both between layers and between genotypes (Fig. 5, legend). In the motor cortex, we detected a significant increase in the density of *Pvalb*-expressing cells in deep layers of *Cyp26b1* CKO mice compared with controls by using paired *t* tests (Fig. 5*D*), but the difference was not significant with repeated-measures two-way ANOVA. More laterally, the putative somatosensory area also did not show a significant difference in density (Fig. 5*E*), suggesting that in the CKO mice, the residual *Cyp26b1* expression in layer 5 of lateral frontal cortex (Fig. 4*K*) might have spared the normal density of *Pvalb*-expressing cells in lateral cortex. In the medial PFC, normal expression of *Cyp26b1* is limited to layer 6, and CKO mice had no residual expression in more superficial layers. In contrast to *Pvalb*-expressing neurons, the density of *Sst*, *Vip* (derived from CGE), or *Lhx6* (a general marker for MGE-derived interneurons)-expressing neurons was not significantly different in the CKO mice compared with controls (Fig. 5*J–R*). Furthermore, in adult brains, *Pvalb*-, *Sst*-, and *Vip*-expressing cells did not show a significant difference between the CKO and control mice (Fig. 5*H*,*I*). Together, these results indicate that transient expression of *Cyp26b1* in layer 6 of medial PFC is specifically required for controlling the development of PV interneurons. This is consistent with a disproportionately high percentage of PV neurons responding to RA compared with SST neurons (Fig. 3). The lack of significant change in the density of *Sst-*, *Vip*-, or *Lhx6*-expressing cells suggests that CYP26B1 does not control the fate specification or the number of each interneuron type, but rather the rate of maturation of PV lineage cells specifically.

### *Cyp26b1* is not expressed in early postnatal medial PFC in the absence of thalamus-PFC connectivity

**Figure 6.**
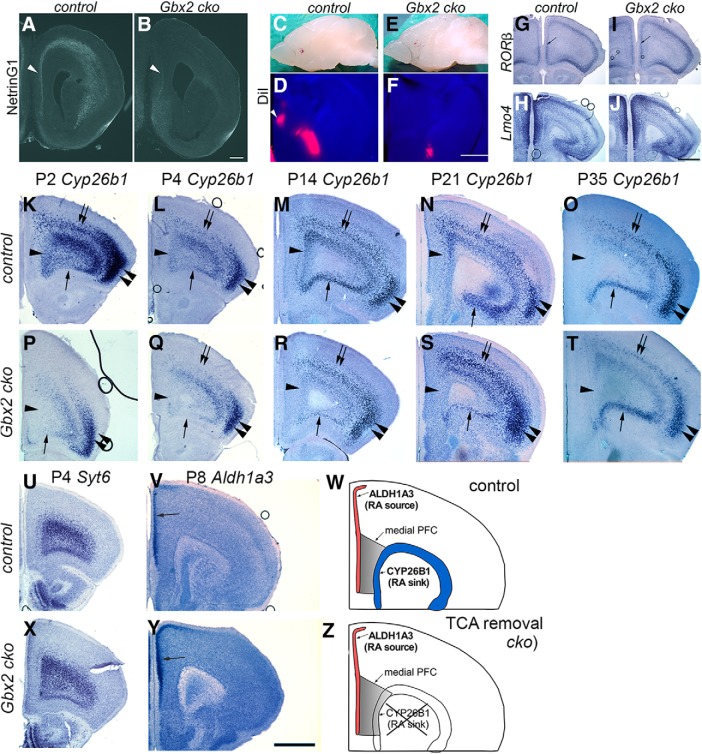
Transient expression of *Cyp26b1* in the PFC does not occur in the absence of thalamus–cortex interactions in *Gbx2* mutant mice. ***A–F***, Thalamus–PFC disconnection in *Gbx2* mutant mice. ***A***, ***B***, Immunostaining for NetrinG1 at E16.5. In control mice, NetrinG1-labeled thalamocortical axons are visible in coronal sections of frontal cortex. Arrowhead in ***A*** shows the medial PFC, where robust labeling is detected. In contrast, NetrinG1 labeling is barely detectable in the frontal cortex of *Gbx2* mutant mice, including the medial PFC (***B***, arrowhead). Scale bar, 200 µm. ***C–F***, DiI labeling at P14. ***C***, ***D***, DiI placement in medial PFC retrogradely labels medial thalamic nuclei in the control brains. ***E***, ***F***, In *Gbx2* mutants, the label is severely reduced, indicating the deficiency of both thalamocortical and corticothalamic projections. ***G–J***, Expression of *RORβ* and *Lmo4* is qualitatively normal in the PFC of *Gbx2* mutant mice at P8. ***G***, ***I***, The expression of *RORβ* in layer 4 is comparable between control (***G***) and *Gbx2* mutant (cko) mice (***I***, arrows). ***H***, ***J***, Laminar expression patterns of *Lmo4* also appear unchanged in *Gbx2* mutants. Scale bar, 1 mm. ***K–Z***, Transient expression of *Cyp26b1* in the PFC does not occur in the absence of thalamus–cortex interactions in *Gbx2* mutant mice. ***K–T***, *in situ* hybridization of frontal sections through PFC at various postnatal stages with a *Cyp26b1* probe. ***K–O***, In control mice (***K–O***), *Cyp26b1* expression starts at P2 in medial (***K***, arrowhead) and ventral (***K***, single arrow) PFC, and continues until P14 (***M***). ***N***, ***O***, At P21, expression in medial PFC is reduced (***N***) and is no longer detectable at P35 (***O***). ***K–O***, In addition to medial and ventral PFC, *Cyp26b1* is also expressed in lateral frontal cortex, including the motor and somatosensory areas (double arrows) and agranular insula (double arrowheads). ***P–T***, In *Gbx2* mutant mice, the expression of *Cyp26b1* is not induced in medial or ventral PFC at P2 as well as at later stages, although ventral PFC does not appear to be affected at P14 and later. ***P–T***, Expression in more superficial layer of lateral cortex (double arrows and double arrowheads) is not affected in *Gbx2* mutant mice. ***U***, ***X***, Expression of the layer 6 marker *Syt6* is not affected in *Gbx2* mutant mice. ***V***, ***Y***, Expression of *Aldh1a3* in layer 2 of medial PFC and anterior cingulate cortex (arrow) is not affected in *Gbx2* mutant mice. Scale bar, 1 mm. ***W***, ***Z***, Summary schematic for this figure.

Because *Cyp26b1* starts to be expressed in medial and ventral PFC when the reciprocal connections between the thalamus and the cortex are being established, we next asked whether the normal expression of *Cyp26b1* depends on this connectivity. In our previous study, thalamus-specific deletion of the homeobox gene *Gbx2* resulted in severe deficiency of thalamocortical and corticothalamic projections in sensory areas (Vue et al., 2013). Similar to sensory cortex, the PFC also showed a significant reduction in the staining of NetrinG1, a marker of thalamocortical axons (Nakashiba et al., 2002; Vue et al., 2013), in *Gbx2* mutant mice at E16.5 (Fig. 6*A*,*B*). Placement of DiI crystals into the medial PFC of *Gbx2* mutants and wild-type mice at P14 revealed that both retrograde labeling of thalamic neurons and anterograde labeling of corticothalamic axons were severely attenuated in *Gbx2* mutants (Fig. 6*C–F*). These results demonstrate a robust reduction of reciprocal connectivity between the thalamus and PFC in *Gbx2* mutant mice.

We then tested whether the expression of *Cyp26b1* in the PFC is altered in *Gbx2* mutant mice. Already at P2, the mutant cortex lacked the expression of *Cyp26b1* in medial and ventral PFC (Fig. 6*K*,*P*), demonstrating that the onset of *Cyp26b1* expression requires thalamocortical interactions. The deficiency of *Cyp26b1* expression continued until P21, when the medial PFC expression of *Cyp26b1* normally started to decline (Fig. 6*N*,*S*). In contrast, the expression of *Cyp26b1* showed no alterations in layer 5 of the lateral frontal cortex, including the motor areas and agranular insula (Fig. 6*K–T*). The layer 6 marker *Syt6* was still highly expressed in the frontal cortex of *Gbx2* mutants (Fig. 6*U*,*X*), making it unlikely that cell loss in layer 6 was the cause of the reduced expression of *Cyp26b1* in *Gbx2* mutants. Lastly, *Aldh1a3*, which normally shows the onset of expression in medial PFC similar to that of *Cyp26b1*, was qualitatively unaffected in *Gbx2* mutants (Fig. 6*V*,*Y*). In summary, transient expression of *Cyp26b1* in layer 6 of medial PFC was dependent on the connections between the thalamus and the cortex (Fig. 6*W*,*Z*). Our results collectively suggest a novel, indirect role of the thalamus in regulating the neocortical interneuron development.

### Lack of thalamus–PFC connectivity results in early aberrancy of radial positioning of MGE-derived interneurons

**Figure 7.**
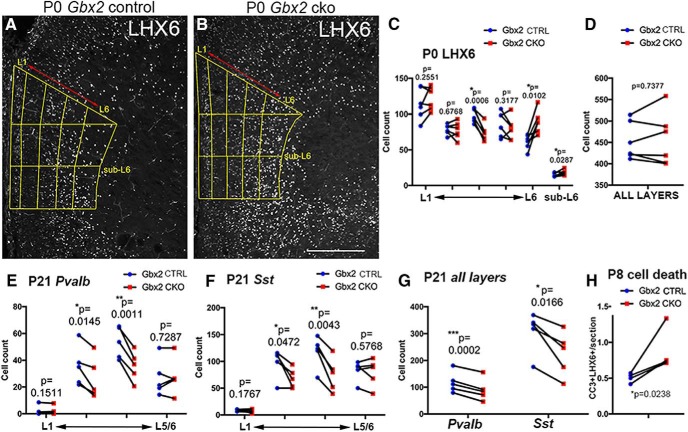
Abnormal radial distribution of MGE-derived interneurons in the medial PFC of neonatal and P21 *Gbx2* mutant mice. ***A***, ***B***, Representative images of immunostaining for LHX6 in medial PFC of wild-type (***A***) and *Gbx2* mutant (***B***) mice at P0. Binning is shown in yellow. Layer 1 (L1) was defined as the cell-sparse layer detected by DAPI staining. Layer 6 (L6) was defined as the layer with TBR1 staining on the same sections (data not shown). The intervening region was equally divided into three layers. Sublayer 6 was defined as the layer below layer 6. Scale bar, 200 μm. ***C***, ***D***, Comparison of LHX6-positive cells in *Gbx2* mutants (red dots) and wild-type littermates (blue dots) in medial PFC at P0. Each line connecting red and blue dots represents a pair of brains analyzed in the same experiment (*n* = 5). ***C*** shows laminar distribution pattern. The *p* values of paired *t* test for individual layer are shown. In repeated-measures two-way ANOVA, the *p* values for layer, pair (between control and knockout), and interactions (between layer and pair) are 0.0001, 0.5950, and 0.0021, respectively. ***D*** shows the total number of *Pvalb*- and *Sst*-expressing neurons in all layers. The *p* values of paired *t* tests are shown. “Sub-L6” was defined as the region below the expression domain of TBR1, which was stained in all immunostaining slides for a reference. *p < 0.05, **p < 0.005, ***p < 0.0005. ***E–G***, Comparison of *Pvalb*-positive and *Sst*-positive cells in *Gbx2* mutants (red dots) and wild-type littermates (blue dots) in medial PFC at P21. Each line connecting red and blue dots represents a pair of brains analyzed in the same experiment (*n* = 5). ***E***, ***F***, Comparison of laminar distribution of *Pvalb*-expressing and *Sst*-expressing neurons, respectively, in *Gbx2* mutant mice and littermate controls. Layer 1 was defined as the cell-sparse layer detected by DAPI staining. The remaining cortical wall was equally divided into three layers. The deepest layer (shown as “L5/6”) contains the entire layer 6 and the deep part of layer 5. ***G***, The total number of *Pvalb*- and *Sst*-expressing neurons in all layers. The *p* values of a paired *t* test for an individual layer are shown. In repeated-measures two-way ANOVA, the *p* values for layer, pair (between control and knockout), and interactions (between layer and pair) are <0.0001, 0.0002, and 0.0001 (*Pvalb*); <0.0001, 0.0247, and 0.0001 (*Sst*), respectively. ***H***, Comparison of the numbers of cleaved caspase 3-positive, LHX6-positive cells in *Gbx2* mutants (red dots), and wild-type littermates (blue dots) in medial PFC at P8. Each line connecting red and blue dots represents a pair of brains analyzed in the same experiment (*n* = 4). Each value is the mean of 7–15 sections. The *p* values of ratio paired *t* tests are shown.

In sensory cortex, thalamocortical afferents affect the development of interneurons by a variety of mechanisms (Sugiyama et al., 2008; Marques-Smith et al., 2016; Tuncdemir et al., 2016; Zechel et al., 2016). However, roles of the thalamus in the development of PFC interneurons are unknown. Hence, we analyzed the distribution of MGE-derived interneurons in medial PFC at P0 and P21. At P0, LHX6-expressing, MGE-derived interneurons showed an altered radial distribution in medial PFC (Fig. 7*A–D*); there was an increase in the density of LHX6-positive cells in layer 6 and below, and a decrease in the middle layers (Fig. 7*C*), while the total density was not significantly different in mutant brains (Fig. 7*D*). These results on the neonatal PFC are remarkably similar to a recent report on sensory and motor cortex of thalamus-specific *Gbx2* mutant mice (Zechel et al., 2016). Thus, there is an early, cortex-wide role of thalamocortical projections in controlling the radial distribution of MGE-derived interneurons. Because the altered neuronal positioning occurred before the onset of *Cyp26b1* expression in medial PFC, these early roles are likely to be independent of the later role of the thalamus in regulating the development of PV interneurons via *Cyp26b1*. At P21, densities of *Pvalb*- and *Sst*- expressing interneurons in medial PFC, specifically in the middle layers and not in the most superficial and the deepest layers, were significantly reduced in *Gbx2* mutant mice (Fig. 7*E–G*). This is consistent with the increased apoptosis of LHX6-expressing cells in *Gbx2* mutants at P8 (Fig. 7*H*), which might reflect the reduced excitatory input onto MGE-derived interneurons in the absence of thalamocortical afferents (Wong et al., 2018).

### Induction of *Cyp26b1* by the thalamus is independent of transmitter release from thalamocortical projection neurons

**Figure 8.**
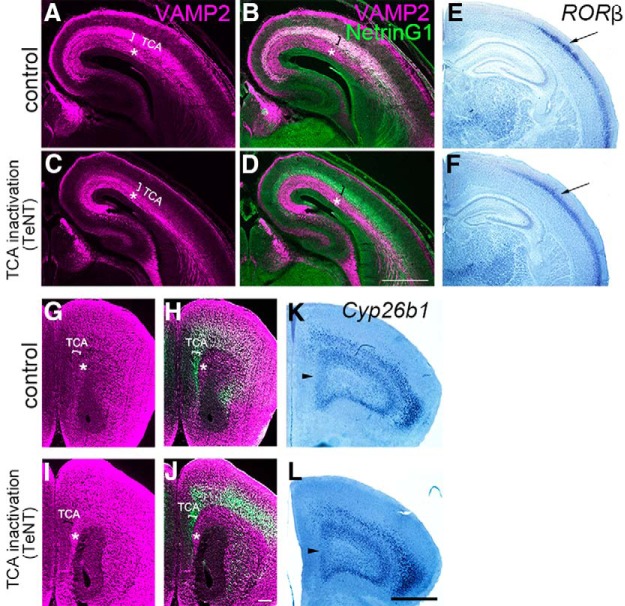
Normal induction of *Cyp26b1* in PFC in mice expressing tetanus toxin light chain in thalamocortical axons. ***A–D***, Immunostaining for VAMP2 on frontal sections of somatosensory cortex at E16.5 control (***A***, ***B***) and mutant mice with ectopic expression of TeNT in thalamic neurons (***C***, ***D***). TeNT expression leads to the deletion of VAMP2, specifically in thalamocortical axons at E16.5. Thalamocortical axons are shown by NetrinG1 staining (***B***, ***D***, green). In control brains, both thalamocortical (bracket, ***A–D***) and corticofugal (asterisk, ***A–D***) axons express VAMP2, whereas in TeNT-expressing mice, VAMP2 staining is specifically diminished in thalamocortical axons (***C***, ***D***, bracket). Scale bar, 500 μm. ***E***, ***F***, Deletion of VAMP2 in thalamocortical axons results in the lack of the characteristic pattern of *RORβ* expression in the barrel field of primary somatosensory cortex at P8 (arrow), similar to the defect found in *Gbx2* mutant mice (Vue et al., 2013). ***G–J***, Immunostaining for VAMP2 on frontal sections of prefrontal cortex at P0 control (***G***, ***H***) and mutant mice with ectopic expression of TeNT in thalamic neurons (***I***, ***J***). Similar to the somatosensory cortex, VAMP2 staining in thalamocortical axons is diminished in TeNT-expressing mice (***I***, ***J***, bracket). Scale bar, 200 μm. ***K***, ***L***, Expression of *Cyp26b1* in medial (arrowhead) and ventral PFC is intact in TeNT-expressing mice (***L***), similar to control (***K***), at P8. Scale bars: ***G***, ***H***, ***I***, ***J***, 200 μm; ***E***, ***F***, ***K***, ***L***, 1 mm.

How does the thalamus control the expression of *Cyp26b1* in layer 6 neurons in medial PFC at early postnatal stages? One likely cue that mediates the role of the thalamus is the transmitter release from thalamocortical axons. To test whether the lack of transmitter release phenocopies the lack of the axon projections, we generated mutant mice in which TeNT is ectopically expressed specifically in thalamocortical projection neurons (Fig. 8). At E16.5, the expression of VAMP2, the cleavage target of TeNT, was dramatically reduced in thalamocortical axons expressing TeNT, while it was retained in corticofugal axons (Fig. 8*A–D*,*G–J*). At P8, the expression of *RORβ* in layer 4 of the primary somatosensory area was altered in TeNT-expressing mice, lacking the characteristic barrel-like pattern (Fig. 8*E*,*F*). This is consistent with a recent study on *vGluT* mutants (Li et al., 2013) and indicates the role of transmitter release in the formation of normal cytoarchitecture of the primary sensory cortex. In the PFC, however, the induction of *Cyp26b1* in the medial and ventral PFC was not qualitatively affected in TeNT-expressing mice (Fig. 8*K*,*L*), implying a unique cellular mechanism that underlies the induction of *Cyp26b1* expression in early postnatal PFC.

## Discussion

**Figure 9.**
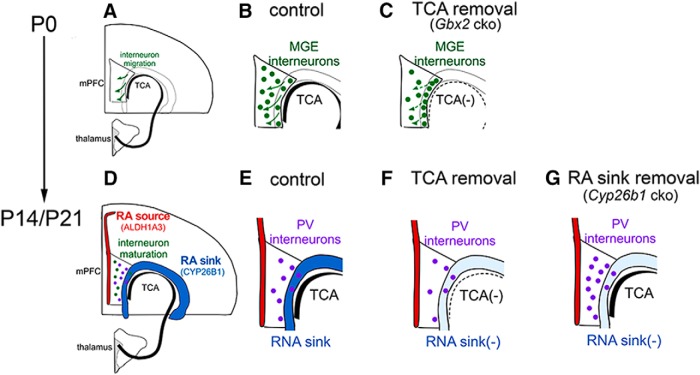
Schematic diagrams of the current finding. ***A–C***, Embryonic roles of thalamocortical axons as observed in neonatal mice. ***A***, Thalamocortical axons reach the medial PFC (mPFC) by E16.5 and control migration of MGE-derived interneurons. ***B***, In normal neonatal mice, MGE-derived interneurons have largely completed tangential migration to the mPFC and have taken proper laminar positioning by radial dispersion (arrows). ***C***, In thalamus-specific *Gbx2* mutant mice, radial positioning of MGE-derived interneurons are aberrant, resulting in their accumulation in layer 6 and below. ***D–G***, Postnatal roles of thalamus–PFC interactions and RA-degrading enzyme CYP26B1 in the development of PV interneurons in the mPFC. ***D***, Early postnatal mPFC is positioned between the source of RA synthesis (layer 2, by ALDH1A3) and the RA-degrading “sink” (layer 6, by CYP26B1). The expression of both enzymes is induced early postnatally, but only *Cyp26b1* is dependent on the connections with the thalamus. The main cell population that responds to RA in postnatal mPFC is PV interneurons, and their development is controlled by CYP26B1. ***E***, In normal postnatal mice, PV neurons mature and start to express *Pvalb* mRNA and PV protein mainly in deep layers of mPFC between P7 and P14. ***F***, In thalamus-specific *Gbx2* mutant mice, *Cyp26b1* is not induced in mPFC. The number of both *Pvalb* and *Sst*-expressing neurons is reduced in the middle layers at least partly due to the earlier defects in radial dispersion (described in ***C***). ***G***, In frontal cortex-specific *Cyp26b1* mutant mice, lack of the RA sink in mPFC leads to an increased number of neurons that express *Pvalb* mRNA or PV protein in deep layers.

In this study, we first demonstrated that *Cyp26b1*, which encodes an RA-degrading enzyme and a critical regulator of retinoid signaling (Duester, 2008; Rhinn and Dollé, 2012), is expressed in developing PFC in a temporally and regionally specific manner. We also showed that PV-expressing interneurons are the major cell population that normally responds to RA via the RAR/RXR receptors. Conditional deletion of *Cyp26b1* in layer 6 of the frontal cortex resulted in an increased density of *Pvalb*-expressing neurons in deep layers of medial PFC during postnatal development. Expression of *Cyp26b1* in the PFC depended on the connections between the cortex and the thalamus, but not on the transmitter release from thalamocortical axons. These results demonstrate a unique regulatory role of the thalamus in postnatal development of PV interneurons in the PFC (Fig. 9*D–G*).

### Roles of RA signaling in postnatal development of the medial PFC

In early embryonic brain, RA controls rostrocaudal patterning of the hindbrain and spinal cord (Maden et al., 1996; Dupé and Lumsden, 2001). Cells in the subventricular zone of the embryonic LGE express the RA-synthesizing enzyme ALDH1A3, and *Aldh1a3*-deficient mice had reduced expression of dopamine receptor D2 (*Drd2*) in nucleus accumbens (Molotkova et al., 2007) and reduced expression of *Gad1* in embryonic GABAergic neurons in the striatum and the cortex (Chatzi et al., 2011), demonstrating a crucial role of RA in the early differentiation of embryonic GABAergic neurons. In contrast, much less is known about the roles of RA signaling in postnatal brain development. Systemic administration of RA into early postnatal mice caused an increased number of calbindin-expressing neurons in the adult cingulate cortex (Luo et al., 2004), implicating a role of RA in early postnatal brain development. However, the role of RA in PFC development and the underlying cellular and molecular mechanisms had remained unknown.

Our current study revealed that in postnatal PFC, a significant subpopulation of PV interneurons are responsive to RA via RAR/RXR receptors, and that the lack of the RA-degrading enzyme CYP26B1 causes an increase in the density of *Pvalb*-expressing cells in medial PFC at P14 and P21. This phenotype should be independent of the proposed earlier roles of RA in tangential migration of GABA neurons (Crandall et al., 2011) because (1) most MGE-derived interneurons have completed the tangential migration to the cortex by birth; (2) *Cyp26b1* is expressed only postnatally in the medial PFC and is not expressed in embryonic MGE or LGE (Fig. 1*M*,*N*); (3) *Syt6-Cre* mice do not cause recombination in MGE-derived interneurons (Fig. 4*A*,*B*); and (4) in *Syt6^Cre/+^; Cyp26b1^flox/flox^* mice, the expression of *Cyp26b1* is not affected in regions outside of the frontal cortex, including the ventral telencephalon (Fig. 4*L–Q*). In addition, the increased density of *Pvalb*-positive neurons in deep layers of medial PFC was not accompanied by their decrease in upper layers. This suggests that the radial dispersion of PV neurons, which follows their tangential migration, was also unaffected in *Cyp26b1* mutants.

In adult brains, *Cyp26b1* mutants no longer showed a significant increase in the density of *Pvalb*-positive neurons in medial PFC (Fig. 5*H*). In addition, the total density of MGE-derived interneurons marked by *Lhx6* did not show a significant change throughout postnatal development. Thus, the most likely role of *Cyp26b1* in the postnatal development of PV neurons in the medial PFC is to slow their rate of maturation by suppressing RA signaling. It is known that neurotrophin (e.g., BDNF) signaling and neuronal activity, likely mediated by NMDA receptors, play a role in the postnatal development of PV neurons (Huang et al., 1999; Itami et al., 2000; Patz et al., 2004; Woo and Lu, 2006; Belforte et al., 2010). Further studies are needed to investigate the relationship between these pathways and RA signaling. It will also be informative to analyze various aspects of maturation of PV neurons including intrinsic electrophysiological properties, formation of perineuronal nets, and changes in gene expression (Okaty et al., 2009; Ueno et al., 2017).

### Universal and area-specific roles of the thalamus in neocortical development

Previous studies have indicated that thalamocortical afferents instruct the establishment of area- and layer-specific gene expression as well as morphologic differentiation of excitatory neurons in primary visual and somatosensory cortex (Chou et al., 2013; Li et al., 2013; Vue et al., 2013; Moreno-Juan et al., 2017), and that some of the effects are dependent on the release of neurotransmitters from thalamocortical axons (Li et al., 2013). In addition to excitatory neurons, both SST and PV interneurons also depend on thalamic afferents for their maturation, likely via glutamatergic synaptic transmission (Sugiyama et al., 2008; Marques-Smith et al., 2016; Tuncdemir et al., 2016). In somatosensory, visual, and motor areas, thalamic afferents also control the radial positioning of MGE-derived interneurons before birth (Zechel et al., 2016). Are these roles universal throughout the cortex or is there area-specific regulation of cortical development by the thalamus? Due to the diverse patterns of gene expression (Jones, 2007; Nagalski et al., 2016; Phillips et al., 2018) and axon projections (Clascá et al., 2012) among different thalamic nuclei and the early regional specification of the cortex before the arrival of thalamic axons (Rash and Grove, 2006; O’Leary et al., 2007; Hoch et al., 2009), it is expected that the nature of interactions with the thalamus varies among different cortical areas.

In fact, the expression of ROR*β* and *Lmo4*, which showed abnormal patterns in primary sensory cortex in the absence of thalamic input (Vue et al., 2013), was not impaired in the medial PFC (Fig. 6*G–J*). Instead, our current study has revealed a frontal cortex-specific regulation of *Cyp26b1* by the thalamus at early postnatal stages. Interestingly, our TeNT model, in which the transmitter release from thalamocortical axons was blocked, was insufficient for recreating the loss of *Cyp26b1* seen in the anatomic deficiency of thalamocortical connectivity. Therefore, the role of the thalamus in inducing *Cyp26b1* expression in medial PFC neurons is likely independent of transmitter release that involves VAMP2 functions.

The current study also revealed an early role of the thalamus in regulating the radial positioning of MGE-derived interneurons in neonatal PFC. The altered positioning of interneurons was similar to the phenotype seen in sensory and motor areas lacking the thalamic input (Zechel et al., 2016). This indicates that the early role of the thalamus in controlling the radial positions of cortical interneurons is shared between many neocortical areas (Fig. 9*A–C*). Importantly, abnormal radial distribution of cortical interneurons was also found in *Lhx6* mutant mice (Liodis et al., 2007), which suggests that the tangential-to-radial switch of interneuron migration in the cortex requires coordination of intrinsic and extrinsic signaling mechanisms during embryonic development.

It is intriguing that in postnatal *Gbx2* mutant mice, even in the absence of normal induction of *Cyp26b1* in medial PFC, the density of *Pvalb*-expressing neurons was decreased, not increased, unlike in *Cyp26b1* mutants. This suggests that the thalamus plays additional roles, including the cell survival, in postnatal development of PV neurons other than by inducing *Cyp26b1.* This possibility is supported by our finding that there is an increased number of cleaved caspase 3-positive, LHX6-expressing cells in *Gbx2* mutants at P8. It will be important to determine whether other known roles of the thalamus in postnatal regulation of the development of PV neurons in sensory cortex (Sugiyama et al., 2008; Marques-Smith et al., 2016; Tuncdemir et al., 2016) also apply to the PFC.

### Functional implications of altered PV neuron development in *Cyp26b1* mutant mice

At the systems level, RA regulates cortical synchrony during sleep (Maret et al., 2005), memory, and cognitive behaviors (Chiang et al., 1998; Aoto et al., 2008; Nomoto et al., 2012). In addition, aberrant RA signaling is associated with multiple psychiatric disorders including schizophrenia, bipolar disorder, and depression in humans (Goodman, 1998; Bremner et al., 2012; Haybaeck et al., 2015; Qi et al., 2015). Thus, understanding how RA functions in early postnatal brain development is important for determining the long-term consequences of the perturbations of this signaling pathway. Our results suggest that RA promotes postnatal development of PV neurons in medial PFC. PV neurons orchestrate activity in local circuits, which leads to synchronous network activity in the gamma band (Cardin et al., 2009; Sohal et al., 2009). Synchronous gamma-band activity in medial PFC is associated with the successful operation of working memory. In a mouse genetic model of schizophrenia that replicates the human 22q11.2 microdeletion syndrome (Karayiorgou et al., 1995), gamma synchrony and working memory performance were impaired (Sigurdsson et al., 2010), so was the development of PV interneurons (Meechan et al., 2012, 2015; Fénelon et al., 2013; Toritsuka et al., 2013). These findings link PV interneuron abnormalities to changes in prefrontal synchrony and working memory impairment in mouse models of neuropsychiatric disorders. Mutation of CYP26B1 in humans is a risk factor for schizophrenia that reaches genome-wide significance (Mistry et al., 2013; Schizophrenia Working Group of the Psychiatric Genomics Consortium, 2014). Therefore, it will be interesting to test whether the aberrant timecourse of PV neuron development in medial PFC causes altered synchrony and impairment of cognitive behaviors in *Cyp26b1* mutant mice.

## Synthesis

Reviewing Editor: Orly Reiner, Weizmann Institute of Science Decisions are customarily a result of the Reviewing Editor and the peer reviewers coming together and discussing their recommendations until a consensus is reached. When revisions are invited, a fact-based synthesis statement explaining their decision and outlining what is needed to prepare a revision will be listed below. The following reviewer(s) agreed to reveal their identity: NONE. Note: If this manuscript was transferred from JNeurosci and a decision was made to accept the manuscript without peer review, a brief statement to this effect will instead be what is listed below.

The authors addressed most of the comments of reviewer 1 and a portion of the comments of reviewer 2 from the journal of neuroscience. The manuscript was sent to an additional reviewer, and the comments are pasted below.

Please address all of the comments prior to resubmission.

This paper convincingly demonstrates the novel finding of elevated RxR activity in PV interneurons, and that a presumed reduction in this activity occurs endogenously due to expression of the Schiz- associated p450 cytochrome 26b1, and that loss of 26b1 results in premature maturation of the PV interneurons. There are many future directions, most mentioned, one that is not is that precicious interneuron maturation would likely effect critical period associated plasticity, although for medial frontal cortex I am not sure what behaviors to test. The 50% colabeling of PV with the RA reporter raises the question of whether it is labeling a defined subgroup. ON P8 the diffuse RA-Lacz labeling --could that be in astrocytes? Line 419--this is consistent with a relationship between activity and interneuron survival p7-p21.--should cite recent paper on mtor signaling, interneuron survival and pyramidal neuron activity from O Marin.

The methods section needs details of how cell counts were done.

In using the term “PFC” to describe cortical areas in rodents, what anatomical boundary does the “P” stand for?

The significance statement is an overstatement. PV interneuron abnormalities are associated with asd, schizophrenia, but a causal link has not been definitely established other than via the folklore created by such overstatements.
